# Supplementary figures and images for: Different gut microbial types were found in captive striped hamsters
Source: PeerJ. 2023 Nov 6;11:e16365. doi: 10.7717/peerj.16365 (PMC10634337; doi:10.7717/peerj.16365)

## Rarefaction curves

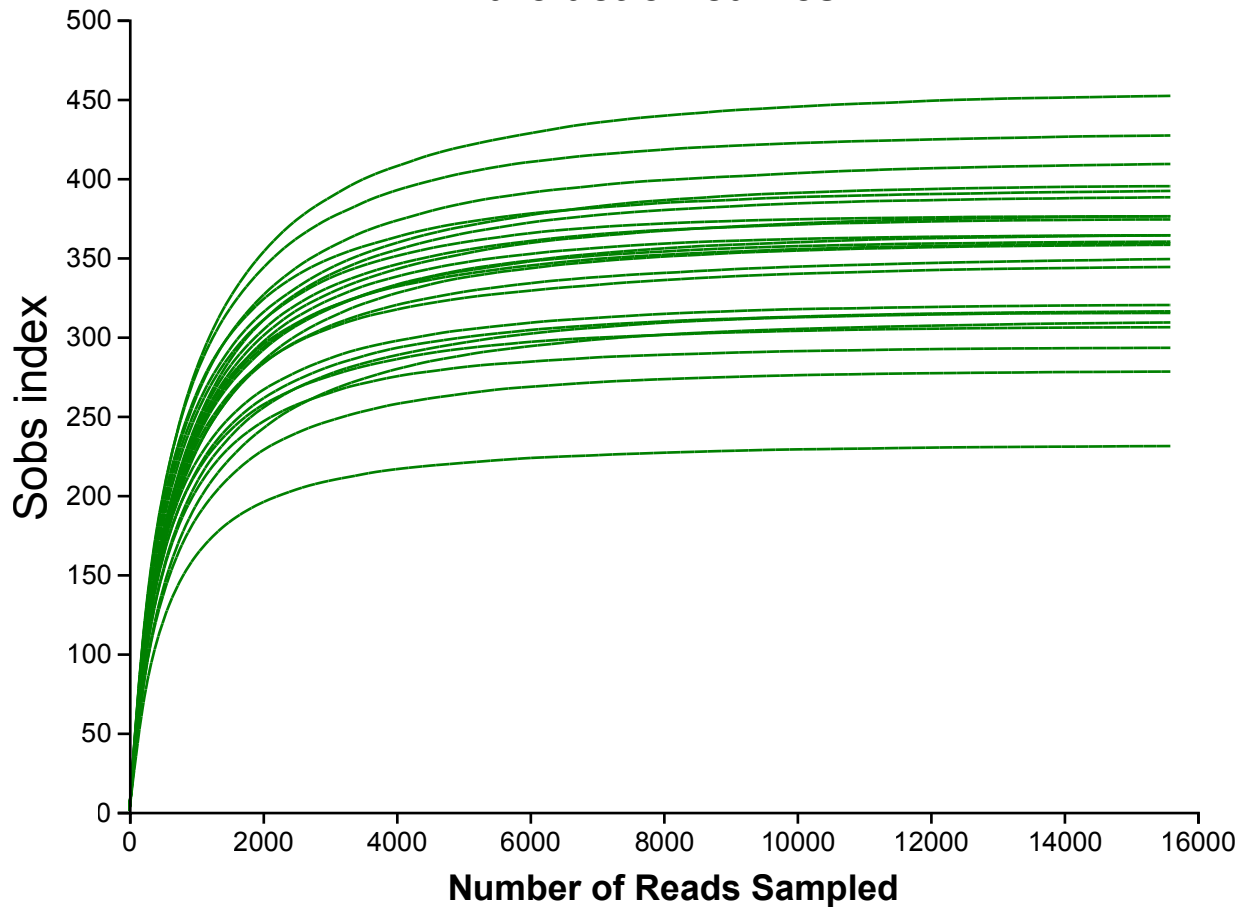

Supplement: Supplemental Information 1 [file peerj-11-16365-s001.pdf]

# Optimal number of clusters

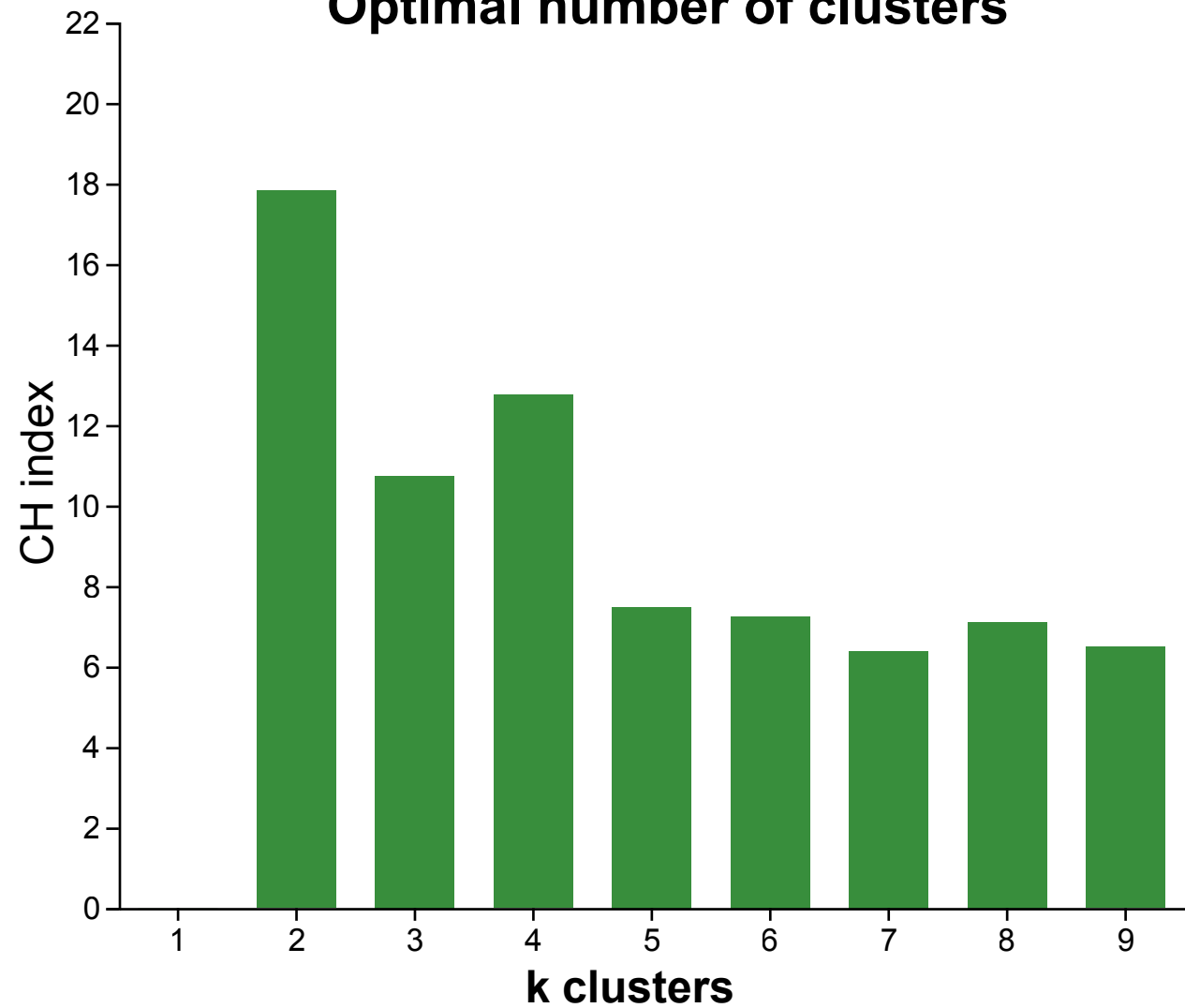

Supplement: Supplemental Information 2 [file peerj-11-16365-s002.pdf]

## Phylum

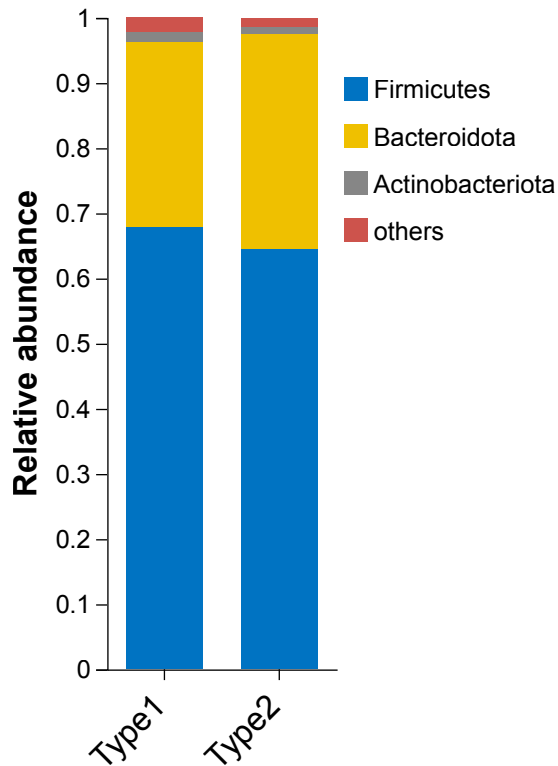

## Family

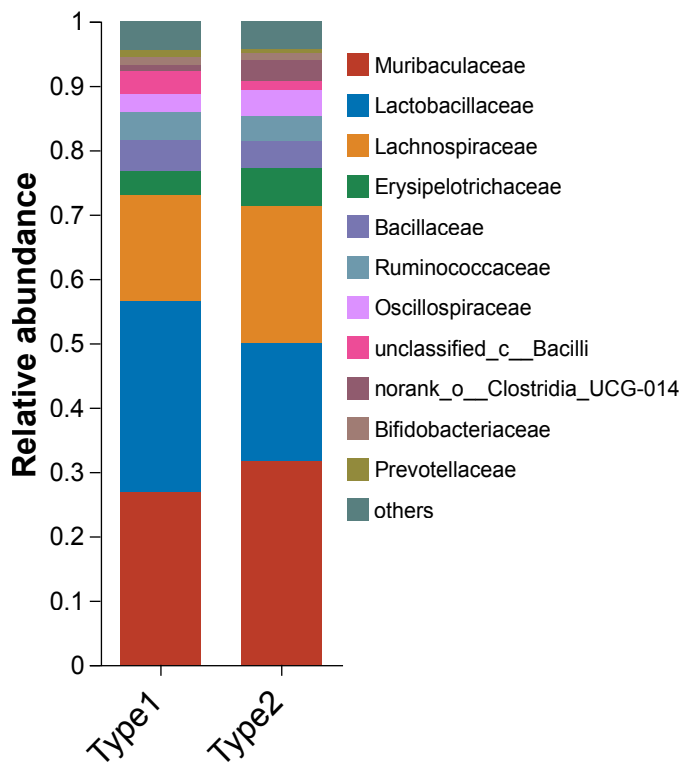

Supplement: Supplemental Information 3 [file peerj-11-16365-s003.pdf]
