# Supplementary material for: Different gut microbial types were found in captive striped hamsters
Source: PeerJ. 2023 Nov 6;11:e16365. doi: 10.7717/peerj.16365 (PMC10634337; doi:10.7717/peerj.16365)
Supplement: Supplemental Information 4 [file peerj-11-16365-s004.pdf]

Table S1 Network indices of the two gut microbial types.

Network indices	Type1	Type2
Total nodes	21	22
Total links	25	36
Positive links	14	21
Negative links	11	15
Average Degree	2.381	3.273
Network Diameter	9	6
Average path length	3.804	2.466
Density	0.119	0.156
Average Clustering Coefficient	0.397	0.563
Total triangles	8	25
